# Imaging characteristics of hepatic metastases from abdominal leiomyosarcoma: A case report

**DOI:** 10.1097/MD.0000000000045580

**Published:** 2025-10-31

**Authors:** Wenyan Du, Xiaoxi Liu, Yiwen Ding, Zekai Zhang, Peng Tian

**Affiliations:** a Department of Clinical Laboratory, Zibo Central Hospital, Zibo, Shandong Province, China; b Department of Ultrasonic, Zibo Central Hospital, Zibo, Shandong Province, China.

**Keywords:** computed tomography, hepatic metastases, leiomyosarcoma, magnetic resonance imaging, ultrasonography

## Abstract

**Rationale:**

Hepatic metastases from abdominal leiomyosarcoma are an extremely rare clinical condition. The multimodal imaging features of this condition have not been systematically described in the current literature, which may lead to delayed diagnosis or misdiagnosis and adversely affect patient prognosis. We herein report a case of hepatic metastases from abdominal leiomyosarcoma that occurred 5 years after surgical resection, and describe in detail its imaging features on ultrasonography, computed tomography (CT) and magnetic resonance imaging (MRI). Through literature review, we aim to enhance clinicians’ ability to diagnose hepatic metastasis from abdominal leiomyosarcoma.

**Patients Concerns:**

A 78-year-old woman presented to our hospital in January 2025 with bilateral lower limb edema. She had undergone abdominal tumor resection 5 years prior. Postoperative pathology confirmed the primary tumor as abdominal leiomyosarcoma.

**Diagnoses:**

Ultrasonography identified multifocal large masses within the hepatic parenchyma. Abdominal CT demonstrated hepatomegaly with multiple heterogeneously hypodense lesions. MRI revealed multiple hypervascular masses in the liver. In conjunction with the patient’s history, these findings suggested hepatic metastases from abdominal leiomyosarcoma, which was subsequently confirmed by ultrasound-guided core needle biopsy of the hepatic mass.

**Interventions:**

Due to advanced age and poor performance status, surgery was contraindicated. Liposomal doxorubicin plus anlotinib was administered as first-line treatment.

**Outcomes:**

The patient completed 6 treatment cycles stably without severe adverse events leading to discontinuation.

**Lessons:**

Hepatic metastases from abdominal leiomyosarcoma are rare but clinically significant. Comprehensive multimodal imaging (ultrasonography, CT, MRI) plays a pivotal role in characterizing these lesions: ultrasound serves as a noninvasive screening tool, while CT and MRI provide detailed anatomical, morphological and vascular information. Histopathological confirmation via ultrasound-guided core needle biopsy is the gold standard for diagnosis. Early detection of hepatic metastases through comprehensive imaging and timely intervention is critical to improving patient outcomes. This case highlights the need for increased awareness of the imaging features of hepatic metastases from abdominal leiomyosarcoma to facilitate early diagnosis and personalized treatment.

## 1. Introduction

Hepatic metastases from abdominal leiomyosarcoma constitute an exceptionally rare subset among secondary hepatic malignancies, representing <0.5% of all liver metastases. Contemporary oncologic data demonstrate a dismal prognosis, with 5-year overall survival rates of 31.8% reported in the literature.^[1]^ Metastatic recurrence demonstrates significant temporal heterogeneity, with documented intervals ranging from 3 months to 27 years postprimary resection.^[2]^ The diagnostic challenge is compounded by the nonspecific nature of early clinical manifestations, resulting in many patients being deemed ineligible for surgical intervention at initial diagnosis. To our knowledge, cases of hepatic metastases from abdominal leiomyosarcoma are rarely reported, with a paucity of detailed imaging characterization. Detailed imaging data of the patient in this case were acquired using ultrasonography, computed tomography (CT), magnetic resonance imaging (MRI), and ultrasound-guided core needle biopsy (CNB). Through integrative analysis of this case, we systematically evaluated the imaging characteristics of hepatic metastases from leiomyosarcoma. These findings could provide evidence-based guidance for early detection of hepatic metastases from abdominal leiomyosarcoma. The main drawback of this case report is the single case and the small sample size. Larger case series and long-term follow-up studies are needed to clarify the distinctive imaging features of hepatic metastases from abdominal leiomyosarcoma.

## 2. Case report

This study has been reviewed and approved by the Ethics Committee of Zibo Central Hospital with the ethics number 2025 Yan No. 151.

**Figure 1.**
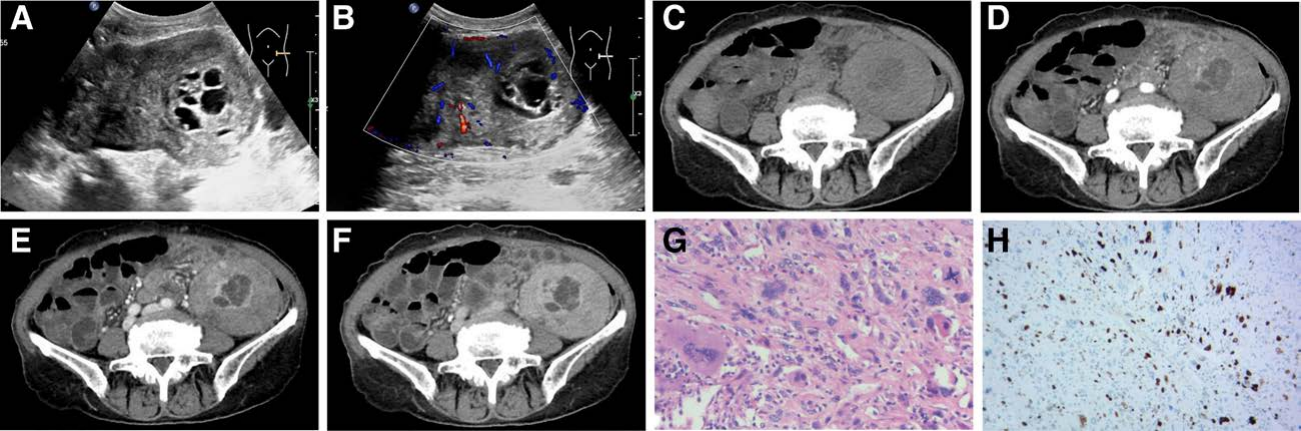
Imaging and pathological findings of the patient in 2020. (A) Ultrasound examination revealed a hypoechoic mass in the lower abdomen. (B) CDFI showed abundant blood flow signals in the solid component. (C) Abdominal CT showed revealed the soft tissue mass within the left lower abdominal quadrant. (D) In the arterial phase, early enhancement was observed at the tumor margin and in some intratumoral areas, whereas a low-density non-enhanced region was noted in the center. (E) During the portal venous phase, the enhanced area extended toward the tumor center, resulting in heterogeneous enhancement. (F) In the delayed phase, persistent heterogeneous enhancement was noted, with a central non-enhanced region remaining. (G) Pathological examination: HE staining (magnitude: 100×). (H) Immunohistochemical result: SMA (+) (magnitude: 100×). CDFI = color Doppler flow imaging, CT = computed tomography, HE = hematoxylin-eosin.

A 78-year-old female was admitted to our hospital in January 2025 with edema of bilateral lower limbs. Blood tests demonstrated hepatic insufficiency (elevated transaminase, hypoalbuminemia) and no abnormalities in renal function. Her previous medical history revealed that the patient presented to our hospital due to the incidental discovery of a lower abdominal mass in February 2020. Ultrasound examination revealed a hypoechoic mass measuring 117 × 62 × 76 mm in the lower abdomen, demonstrating clear boundaries, irregular shape, uneven echo, and irregular anechoic area (Fig. 1A). Color Doppler flow imaging (CDFI) showed abundant blood flow signals in the solid component (Fig. 1B). Abdominal CT revealed the soft tissue mass within the left lower abdominal quadrant with regular shape and uneven density, and the area of the maximum cross-section was approximately 82 × 70 mm (Fig. 1C). The enhanced imaging demonstrates heterogeneous enhancement with a central nonenhancing necrotic component (Fig. 1D–F). The CT equipment used was the Toshiba Aquilion One. This scanner employed a multi-phase enhanced scanning protocol. The contrast agent was ioversol 320, administered at a dosage of 90 mL and an injection rate of 3.0 mL/s. The imaging findings were consistent with the characteristics of malignant tumors. Subsequently, the patient underwent abdominal tumor resection in our hospital. Postoperative histopathological examination revealed a spindle cell tumor originating from the abdominal soft tissues (tumor size: 12 × 8 × 5.5 cm) with cellular atypia and focal necrosis (Fig. 1G). Immunohistochemistry revealed Vimentin:(+); SMA:(+); desmin:(+); CD10:focal(+); P16:(+) and S of 10%; CKAE1/AE3: (−); WT-1: (−); S-100: (−); CD117: (−); Dog-1: (−); CD34: (−); STAT6: (−); bcl-2: (−); CD99: (−); CD68: (−); ALK: (−); Myoglobin: (−); Alpha-statin: (−) (Fig. 1H). The histopathological diagnosis of abdominal leiomyosarcoma was confirmed through immunohistochemical studies. Serum tumor marker analysis demonstrated negative results for carcinoembryonic antigen (CEA), alpha-fetoprotein (AFP), and carbohydrate antigen 19-9 (CA19-9), with all values within normal reference ranges (CEA = 1.6 ng/mL, AFP = 4.98 ng/mL, CA19-9 = 20.7 U/mL). Considering factors such as age and weak physique, doxorubicin liposome single-drug chemotherapy was given to the patient, and postoperative adjuvant radiotherapy was performed after 2 cycles of chemotherapy. Given the patient’s advanced age, the multidisciplinary tumor board recommended liposomal doxorubicin monotherapy. Following 2 cycles of chemotherapy, the patient underwent rigorous surveillance comprising abdominal ultrasonography and thoracic CT scans at 3-month intervals. Over a 24-month follow-up period, serial imaging demonstrated no evidence of metastatic progression. All ultrasound examinations and CT examinations were discontinued in October 2022 because the patient showed no symptoms of physical discomfort.

**Figure 2.**
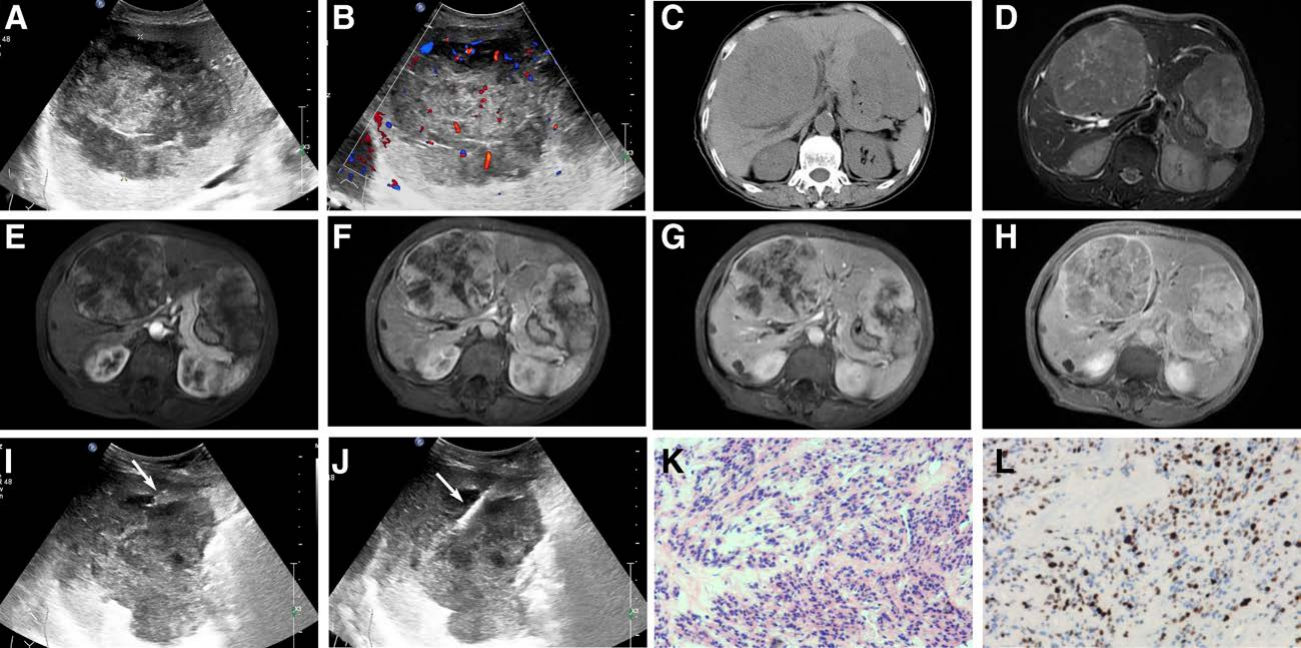
Imaging and pathological findings of the patient in 2025. (A) Ultrasound examination revealed multiple huge hypoechoic masses in the liver. (B) CDFI showed abundant blood flow signals both around and within the tumor. (C) Abdominal CT revealed hepatomegaly with multiple hypodense hepatic lesions. (D) On T2-weighted images, multiple well-defined lesions were observed, demonstrating heterogeneous high signal intensity. (E) In the early arterial phase, peripheral thin rim-shaped enhancement was noted with overall weak and heterogeneous enhancement. (F) In the late arterial phase, the peripheral enhancement extended centripetally with increased enhancement intensity, manifesting as map-like enhancement. (G) In the portal venous phase, the enhanced area gradually expanded centripetally, showing heterogeneous progressive enhancement. (H) In the delayed phase, the enhanced area further expanded or remained stable, with the lesions exhibiting overall heterogeneous high signal intensity. (I) The 16G introducer needle (arrow) transmitted at the leading edge of the liver tumor. (J) The18G semiautomatic biopsy needle (arrow) transmitted in the liver tumor. (K) Pathological results: HE staining (magnitude: 10 × 10). (L) Immunohistochemical result shows Desmin (+) (magnitude: 10 × 10). CDFI = color Doppler flow imaging, CT = computed tomography, HE = hematoxylin-eosin.

Therefore, ultrasound reexamination was suggested during this clinical encounter due to her previous medical history and hepatic insufficiency. Ultrasound examination revealed multiple huge hypoechoic masses in the liver with clear boundaries, irregular shapes, less homogeneous internal echoes, and peripheral hypoechoic halos, the largest of which measured approximately 115 × 91 mm (Fig. 2A). CDFI showed abundant blood flow signals both around and within the tumor (Fig. 2B). Abdominal CT revealed hepatomegaly with multiple hypodense hepatic lesions demonstrating clear boundaries and heterogeneous internal density, the largest lesion measured 111 × 94 mm (Fig. 2C). Enhanced CT was advised to further evaluate the lesion characteristics and confirm the diagnosis. MRI revealed multiple hepatic parenchymal lesions with prolonged T1 and T2 signal intensities, demonstrating well-defined margins and heterogeneous signal intensity, the largest lesion measured 109 × 91 mm (Fig. 2D). Dynamic enhancement scan showed gradual heterogeneous enhancement (Fig. 2E–H). The magnetic resonance equipment used was a GE Signa HDxt (3.0T). The contrast agent was gadopentetic acid injection, administered at a dosage of 15 mL and an injection rate of 4.5 mL/s. Based on the medical history, the tumor was considered highly likely to be metastatic.

Subsequently, we performed an ultrasound-guided CNB of the liver mass and obtained 3 cores of liver tissue using a coaxial system comprising a 16G introducer needle (Fig. 2I) and an 18G semiautomatic biopsy needle (Fig. 2J). Pathological examination revealed malignant tumors in 3 cores of liver tissue (Fig. 2K). Immunohistochemistry revealed Vimentin: (+); SMA: (+); desmin: (+); CD10: (−); P16: (+) S of 45%; CD34: Blood vessel (+); STAT6: Pulp (+); CKAE1/AE3: (−); WT-1: (−); S-100: (−); CD117: (−); Dog-1: (−); ALK: (−); Myoglobin: (−); MelanA: (−); ER: (−); PR: focal (+); C-erbB-2: (0); P53: (−); Ki-67: (+) S of 45% (Fig. 2L). Based on immunohistochemical findings and the patient’s medical history, the diagnosis of hepatic metastases from abdominal leiomyosarcoma was confirmed. Serum tumor marker analysis demonstrated negative results for carcinoembryonic antigen (CEA), alpha-fetoprotein (AFP), and carbohydrate antigen 19-9 (CA19-9), with all values within normal reference ranges (CEA = 1.7 ng/mL, AFP = 3.91 ng/mL, CA19-9 = 19.6 U/mL). Given the patient’s advanced age and compromised clinical status precluding surgical resection, liposomal doxorubicin combined with anlotinib was initiated as palliative systemic therapy.

## 3. Discussion

Leiomyosarcoma is a rare malignant neoplasm of smooth muscle origin, classified as a soft tissue sarcoma. Histologically, it demonstrates tumor cells with definitive smooth muscle differentiation, typically exhibiting spindle-shaped morphology. Leiomyosarcoma accounts for approximately 15% of adult sarcomas. Its etiology remains unclear and may be associated with genetic predisposition, environmental exposures, radiation history, and other factors.^[3]^ Leiomyosarcoma most frequently arises in the retroperitoneum, gastrointestinal tract, genitourinary tract, and blood vessels (e.g., the inferior vena cava) or surrounding soft tissues. Among these sites, the retroperitoneum is the most common, accounting for approximately 50% of all leiomyosarcomas. It predominantly occurs in middle-aged and elderly individuals aged 40 to 70 years, with a higher incidence in women than in men.^[4]^ Leiomyosarcoma is characterized by aggressive growth, with early clinical symptoms often subtle and easily overlooked. The prognosis remains poor, determined primarily by tumor location, size, histological grade, and completeness of resection. Complete surgical resection remains the primary treatment, but it is difficult because the tumor often invades the surrounding tissue. Laitinen et al reported that the 5-year overall survival rate after leiomyosarcoma was 28% to 40%.^[5]^

Metastasis is common in patients with leiomyosarcoma, occurring primarily via hematogenous spread. In contrast, implantation metastasis and lymphatic dissemination are rarely observed.^[6]^ The pattern of metastasis in leiomyosarcoma correlates strongly with the venous drainage pattern of the primary tumor site. Leiomyosarcoma originating in the extremities and trunk most frequently metastasizes to the lungs, while leiomyosarcoma arising in the gastrointestinal tract commonly disseminate to the liver. The principle is that venous return from the extremities and trunk primarily flows into the right heart via the superior and inferior venae cavae, ultimately reaching the lungs through the pulmonary artery. These intricate capillary beds within the pulmonary vasculature provide an ideal microenvironment for tumor cell retention and proliferation. The venous drainage from the gastrointestinal tract predominantly drains to the liver through the portal vein system. Structural features of the liver’s dual blood supply create a permissive microenvironment where circulating tumor cells become mechanically trapped and subsequently proliferate. The metastatic characteristics of leiomyosarcoma suggest that the imaging examination of the liver and lungs should be given priority in postoperative follow-up protocols.

In our study, foreign literatures of hepatic metastasis from leiomyosarcoma were retrieved, and summarized in Table 1.^[7–11]^ Six cases are presented in Table 1, 3 of which underwent ultrasound examination. The ultrasound findings revealed multiple hypoechoic lesions in the liver with clear boundaries and uneven echoes. Larger tumors were frequently associated with partial cystic degeneration. CDFI demonstrated abundant blood flow signals both around and within the tumor. The ultrasonographic characteristics observed in this study are largely consistent with the findings reported in the literature, as summarized in Table 1. Contrast-enhanced ultrasound (CEUS) is valuable in differentiating benign and malignant liver tumors.^[12]^ Malignant tumors typically demonstrate hyperenhancement during the arterial phase, followed by hypoenhancement in the portal phase and delayed phases. The study by Sun et al (summarized in Table 1) corroborates these imaging features, thereby verifying the clinical utility of contrast-enhanced ultrasound (CEUS) in diagnosing hepatic malignancies. All patients included in Table 1 underwent CT scan of the liver. CT features revealed multiple hypodense lesions with well-defined margins. The enhancement pattern was characterized by rapid arterial hyperenhancement, followed by decreased attenuation in the portal venous and delayed phases. The CT findings in this case correlated with the imaging patterns documented in Table 1. However, contrast-enhanced MRI was prioritized in this study; therefore, additional contrast-enhanced CT examinations were not conducted.

**Table 1:** Imaging features of hepatic metastasis from leiomyosarcoma reported in previous literature.

No.	First authors (PubMed unique identifier, year)	Number of cases	Primary site of the tumors	Ultrasonographic characteristics	CT features	MRI findings
1	Sun et al^[7]^ (PMID: 37899675,2023)	1	Small intestinal	Multiple hypoechoic nodules of different sizes; clear borders; regular morphology; inhomogeneous internal echogenicity. CDFI: Peripheral blood flow signals were seen. CEUS showed rapid inhomogeneous hyperenhancement in the arterial phase, while inhomogeneous hyperenhancement persisted in the portal and delayed phases.	Multiple low-density foci with clear boundaries; regular shapes; uneven internal density. During the arterial phase, the lesions demonstrated rapid heterogeneous hyperenhancement. The delayed phase revealed heterogeneous hypoenhancement.	Not reported
2	Alnaqbi et al^[8]^ (PMID: 39606495,2024)	1	Pancreas	Not reported	Multiple low-density lesions with clear borders, the largest of which is located in segment II measuring 8 × 8 cm.	Smooth margins; markedly hyperintense signal on T2-weighted images; homogeneous low signal intensity on T1-weighted images.
3	Mizoshiri et al^[9]^ (PMID: 29718861, 2018)	2	Limbs	Not reported	Multiple low-density lesions; clear borders.	Multiple slightly longer T1 and slightly longer T2 signal; clear boundaries; uneven enhancement pattern.
4	Yoshizawa et al^[10]^ (PMID: 37022631, 2023)	1	Inferior vena cava	Not reported	Multiple low-density foci; clear boundaries; regular shape.	Multiple hypointense nodules in the hepatobiliary phase of Gd-EOB-DTPA-enhanced MRI; well-defined margins
5	Ioanițescu et al^[11]^ (PMID: 34762725, 2022)	1	Spleen	Enlarged liver with multiple hypoechoic tumors; clear margins; CEUS revealed arterial phase iso-hypoenhancement of the liver lesions with rapid washout.	multiple hypodense lesions; well-defined margins.	Not reported

Soyer et al^[13]^ described the MRI characteristics of hepatic metastases from leiomyosarcoma in a cohort of 16 patients. MRI findings demonstrated well-defined margins, markedly hyperintense signals on T2-weighted imaging, and heterogeneous enhancement patterns in 13 of 16 tumors (81.25%). Four patients in Table 1 underwent hepatic MRI evaluation. The predominant MRI characteristics included a clear boundary, T2-weighted hyperintensity, and heterogeneous enhancement patterns.

Larger tumors frequently exhibit central nonenhancing regions, representing necrotic zones secondary to rapid tumor proliferation and inadequate vascularization. These necrotic regions are a typical feature of malignant tumors. The MRI findings observed in this case were basically consistent with imaging characteristics documented in Table 1.

Ultrasound-guided CNB of hepatic masses is a safe and efficacious technique for obtaining pathological specimens, enabling definitive histological diagnosis of space-occupying liver lesions. The most frequent complication associated with ultrasound-guided CNB of hepatic lesions is needle tract bleeding. In this case, a 16G introducer puncture needle was advanced into the liver under ultrasound guidance, with its tip penetrating at least 2 cm beyond the liver capsule. The assistant then withdrew the needle core, and an 18G semiautomatic biopsy needle was inserted through the introducer needle. After 3 cores of liver tissue were obtained via biopsy, gelatin sponge particles were mixed with 3 mL of normal saline and injected into the liver through the introducer needle, while simultaneously withdrawing the needle slowly from the hepatic parenchyma. Gelatin sponge particles are porous, absorbent materials with high fluid-absorbing capacity. They absorb blood and other fluids from the puncture site, subsequently expanding to occlude the needle tract, thereby achieving hemostasis.^[14]^ The use of coaxial puncture technique combined with gelatin sponge particles during ultrasound-guided CNB of liver masses can significantly reduce the risk of needle tract bleeding. This approach has gained widespread recognition among clinicians for its demonstrated efficacy and holds substantial value for clinical adoption. Guo et al^[15]^ reported the application of a mixed embolic agent composed of gelatin sponge particles and thrombin during CNB of the spleen, which demonstrated effective hemostatic outcomes. Immunohistochemical analysis plays a critical role in the diagnostic evaluation and differentiation of metastatic leiomyosarcoma to the liver.^[16]^ As summarized in Table 1, immunohistochemical findings were available for all 6 patients. The main immunohistochemical characteristics demonstrated positivity for SMA, desmin, vimentin, and h-caldesmon, whereas CD117, DOG1, and S100 were negative. The immunohistochemical examination of this case was highly consistent with these features.

At present, surgical resection remains the primary therapeutic approach for hepatic metastases from leiomyosarcoma, with complete excision of lesions significantly improving patient survival. Smolle et al^[17]^ demonstrated that patients with metastatic leiomyosarcoma, particularly those with liver involvement, achieve prolonged survival through radical surgical resection. Lang et al^[18]^ further supported this evidence, showing that surgical resection provides superior long-term outcomes compared to chemotherapy or transcatheter arterial chemoembolization (TACE) in managing hepatic metastases from leiomyosarcoma. However, the early symptoms of liver metastasis of abdominal leiomyosarcoma lack specificity and are easily overlooked, often resulting in missing the optimal timing for surgical resection by the time of diagnosis. Therefore, early detection and diagnosis are crucial, which can enable more patients to undergo effective surgical resection and thus improve the survival rate.

Furthermore, the feasibility of surgical intervention is frequently constrained by multiple clinical factors, such as patient age, comorbidities, hepatic functional reserve, and the number, size, and anatomical distribution of metastatic lesions. Many patients are no longer surgical candidates at the time of liver metastasis diagnosis. TACE is a widely utilized minimally invasive therapy for hepatic tumors.^[19]^ Compared to systemic chemotherapy, TACE offers distinct advantages: 1. Achieving significantly higher intratumoral drug concentrations; 2. Minimizing systemic toxicity of chemotherapeutic agents; 3. Occluding tumor vascular supply through embolization; 4. Inducing selective tumor cell necrosis via targeted drug delivery; 5. Allowing repeated administration for residual or recurrent intrahepatic lesions. Hara et al^[20]^ described a case of duodenal leiomyosarcoma with multifocal hepatic metastases, in which the patient underwent repeated TACE sessions following primary tumor resection, achieving an overall survival of 4 years and 9 months. This evidence supports TACE as an effective palliative modality for unresectable hepatic metastases from leiomyosarcoma.

## 4. Conclusion

The liver is a common site for leiomyosarcoma metastasis. Comprehensive multimodal imaging (ultrasonography, CT, MRI) plays a pivotal role in characterizing these lesions: ultrasound serves as a noninvasive screening tool, while CT and MRI provide detailed anatomical, morphological, and vascular details. Histopathological confirmation via ultrasound-guided CNB is the gold standard for diagnosis. Surgical resection is the preferred treatment for liver metastases, as it significantly improves long-term survival; for inoperable patients, TACE serves as a palliative therapy. Notably, studies on the imaging features of hepatic metastases from abdominal leiomyosarcoma are of great significance for the early diagnosis of patients. In-depth research on the imaging characteristics of this disease can provide a scientific basis for early detection and timely intervention, thereby improving patients’ quality of life.

## Author contributions

**Investigation:** Zekai Zhang.

**Methodology:** Yiwen Ding.

**Resources:** Xiaoxi Liu.

**Supervision:** Peng Tian.

**Writing – original draft:** Wenyan Du.

**Writing – review & editing:** Peng Tian

## References

[1] Marudanayagam R Sandhu B Perera MT. Liver resection for metastatic soft tissue sarcoma: an analysis of prognostic factors. Eur J Surg Oncol. 2011;37:87–92. 21163386 10.1016/j.ejso.2010.11.006

[2] Grimme FAB Seesing MFJ van Hillegersberg R.; On behalf of the Dutch Liver Surgery Working Group. Liver resection for hepatic metastases from soft tissue sarcoma: a nationwide study. Dig Surg. 2019;36:479–86. 30253419 10.1159/000493389 PMC6878742

[3] Mankin HJ Casas-Ganem J Kim JI Gebhardt MC Hornicek FJ Zeegen EN. Leiomyosarcoma of somatic soft tissues. Clin Orthop Relat Res. 2004;421:225–31. 10.1097/01.blo.0000119250.08614.82 15123952

[4] Devaud N Vornicova O Abdul Razak AR. Leiomyosarcoma: current clinical management and future horizons. Surg Oncol Clin N Am. 2022;31:527–46. 35715148 10.1016/j.soc.2022.03.011

[5] Mäkelä J Kiviniemi H Laitinen S. Prognostic factors predicting survival in the treatment of retroperitoneal sarcoma. Eur J Surg Oncol. 2000;26:552–5. 11034804 10.1053/ejso.2000.0945

[6] Lacuna K Bose S Ingham M Schwartz G. Therapeutic advances in leiomyosarcoma. Front Oncol. 2023;13:1149106. 36969049 10.3389/fonc.2023.1149106 PMC10031121

[7] Sun J Li G Huo X Fang N Wang X Xu W. Contrast-enhanced ultrasonography for diagnosis of small intestinal leiomyosarcoma with hepatic metastasis: a clinical report of one case and review of the literature. Curr Med Imaging. 2024;20:1–7. 10.2174/0115734056243285230925001213 37899675

[8] Alnaqbi S Kumar P Bin Sumaida A Shanbhag NM Ahmad AZ Balaraj K. Pancreatic leiomyosarcoma with multi-organ metastases: a rare case. Cureus. 2024;16:e72520. 39606495 10.7759/cureus.72520 PMC11600098

[9] Mizoshiri N Shirai T Terauchi R. Hepatic metastases from primary extremity leiomyosarcomas: two case reports. Medicine (Baltimore). 2018;97:e0598. 29718861 10.1097/MD.0000000000010598 PMC6392636

[10] Yoshizawa K Ohno Y Kurata T. Primary leiomyosarcoma of the inferior vena cava in a pediatric case: a case report and literature review. Surg Case Rep. 2023;9:52. 37022631 10.1186/s40792-023-01630-x PMC10079787

[11] Ioanițescu ES Grasu M Toma L. Primary splenic leiomyosarcoma – case report and literature review. Med Ultrason. 2022;24:114–6. 34762725 10.11152/mu-3325

[12] Li H Li J. Application of real-time contrast-enhanced ultrasound in differential diagnosis of liver malignancies. Can J Physiol Pharmacol. 2019;97:341–4. 30508395 10.1139/cjpp-2018-0404

[13] Soyer P Riopel M Bluemke DA Scherrer A. Hepatic metastases from leiomyosarcoma: MR features with histopathologic correlation. Abdom Imaging. 1997;22:67–71. 9000359 10.1007/s002619900142

[14] Yang X Cheng HT Huang Y. Safety and efficacy of tract embolization using gelatin sponge particles in reducing pneumothorax after CT-guided percutaneous lung biopsy in patients with emphysema. BMC Pulm Med. 2024;24:329. 38982416 10.1186/s12890-024-03125-3 PMC11232318

[15] Guo RQ Li XG. Seven case reports on the prevention of hemorrhage after percutaneous computed tomography-guided core-needle biopsy of the spleen. J Cancer Res Ther. 2020;16:1182–5. 33004768 10.4103/jcrt.JCRT_815_19

[16] Okamoto M Matsuoka M Soma T. Metastases of soft tissue sarcoma to the liver: a historical cohort study from a hospital-based cancer registry. Cancer Med. 2020;9:6159–65. 32648686 10.1002/cam4.3304 PMC7476817

[17] Smolle MA Leithner A Bernhardt GA. Abdominal metastases of primary extremity soft tissue sarcoma: a systematic review. World J Clin Oncol. 2020;11:74–82. 32133276 10.5306/wjco.v11.i2.74 PMC7046921

[18] Lang H Nussbaum KT Kaudel P Frühauf N Flemming P Raab R. Hepatic metastases from leiomyosarcoma: a single-center experience with 34 liver resections during a 15-year period. Ann Surg. 2000;231:500–5. 10749609 10.1097/00000658-200004000-00007 PMC1421024

[19] Sullivan KL. Hepatic artery chemoembolization. Semin Oncol. 2002;29:145–51. 10.1053/sonc.2002.31671 11951212

[20] Hara T Wada I Kajihara S Mizuta T Yamamoto K Sakai T. Case report: a long-term survivor of jejunal leiomyosarcoma with liver metastasis: effective transcathetel arterial embolization for hepatic metastatic foci. J Gastroenterol Hepatol. 1998;13:620–3. 9715406 10.1111/j.1440-1746.1998.tb00700.x

